# Nanoemulsions of *Satureja montana* Essential Oil: Antimicrobial and Antibiofilm Activity against Avian *Escherichia coli* Strains

**DOI:** 10.3390/pharmaceutics13020134

**Published:** 2021-01-21

**Authors:** Federica Rinaldi, Linda Maurizi, Antonietta Lucia Conte, Massimiliano Marazzato, Alessandro Maccelli, Maria Elisa Crestoni, Patrizia Nadia Hanieh, Jacopo Forte, Maria Pia Conte, Carlo Zagaglia, Catia Longhi, Carlotta Marianecci, Maria Grazia Ammendolia, Maria Carafa

**Affiliations:** 1 Dipartimento di Chimica e Tecnologie del Farmaco, Sapienza Università di Roma, Piazzale Aldo Moro 5, 00185 Roma, Italy; federica.rinaldi@uniroma1.it (F.R.); alessandro.maccelli@uniroma1.it (A.M.); mariaelisa.crestoni@uniroma1.it (M.E.C.); patrizianadia.hanieh@uniroma1.it (P.N.H.); jacopoforte96@gmail.com (J.F.); maria.carafa@uniroma1.it (M.C.); 2 Dipartimento di Sanità Pubblica e Malattie Infettive, Sapienza Università di Roma, Piazzale Aldo Moro 5, 00185 Roma, Italy; lindamaurizi92@gmail.com (L.M.); antoniettalucia.conte@uniroma1.it (A.L.C.); massimiliano.marazzato@uniroma1.it (M.M.); mariapia.conte@uniroma1.it (M.P.C.); carlo.zagaglia@uniroma1.it (C.Z.); 3 Centro Nazionale Tecnologie Innovative in Sanità Pubblica, Istituto Superiore di Sanità, Viale Regina Elena, 299, 00161 Rome, Italy; maria.ammendolia@iss.it

**Keywords:** *Satureja montana* L., essential oils, *Escherichia coli*, nanoformulation, nanoemulsions, antibacterial activity, antibiofilm activity, high resolution mass spectrometry

## Abstract

*Satureja montana* essential oil (SEO) presents a wide range of biological activities due to its high content of active phytochemicals. In order to improve the essential oil’s (EO) properties, oil in water nanoemulsions (NEs) composed of SEO and Tween-80 were prepared, characterized, and their antimicrobial and antibiofilm properties assayed against *Escherichia coli* strains isolated from healthy chicken. Since surfactant and oil composition can strongly influence NE features and their application field, a ternary phase diagram was constructed and evaluated to select a suitable surfactant/oil/water ratio. Minimal inhibitory concentration and minimal bactericidal concentration of NEs, evaluated by the microdilution method, showed that the SEO NE formulation exhibited higher inhibitory effects against planktonic *E. coli* than SEO alone. The quantification of biofilm production in the presence of NEs, assessed by crystal violet staining and scanning electron microscopy, evidenced that sub-MIC concentrations of SEO NEs enable an efficient reduction of biofilm production by the strong producer strains. The optimized nanoemulsion formulation could ensure food safety quality, and counteract the antibiotic resistance of poultry associated *E. coli,* if applied/aerosolized in poultry farms.

## 1. Introduction

*Escherichia coli* is a Gram-negative, facultative anaerobic commensal of the vertebrate gut. These bacteria are usually harmless, however, some *E. coli* strains, through acquisition of various virulence factors by the horizontal transfer of plasmids, pathogenicity islands, transposons, and bacteriophages, have gained the ability to cause a variety of enteric diseases, as well as infections at extraintestinal sites: urinary tract, prostate, bloodstream, and others [1,2].

*E. coli* pathotypes that cause intestinal infections have been classified into six different groups: enterotoxigenic *E. coli* (ETEC), enteropathogenic *E. coli* (EPEC), enterohemorrhagic *E. coli* (EHEC), enteroinvasive *E. coli* (EIEC), diffuse adherent *E. coli* (DAEC), and enteroaggregative *E. coli* (EAEC). In addition, invasive adherent *E. coli* (AIEC), associated with Crohn’s disease, belongs to the class of intestinal pathogens [3,4]. Pathogenic *E. coli* that do not induce entero-diarrheal diseases are defined as ExPEC, and include uropathogenic *E. coli* (UPEC), neonatal meningitis-associated *E. coli* (NMEC), sepsis-causing *E. coli* (SEPEC), and avian pathogenic *E. coli* (APEC) [5]. Potential reservoirs for the extraintestinal pathogen *E. coli* (ExPEC) include the human intestinal tract and various non-human reservoirs, such as companion animals, food animals, retail meat products, sewage, and other environmental sources [6].

Different studies have reported that poultry products may represent a source of ExPEC [7,8], and that poultry meat exhibits the highest levels of *E. coli* contamination, and which contains strains often more extensively multidrug resistant than *E. coli* recovered from the meat of other livestocks. The increased poultry meat consumption worldwide could have contributed to the emergence of extraintestinal infections in humans [9]. Similarly to human ExPEC, *E. coli* that cause extraintestinal infections in chickens often originate from the intestines, where they can have a commensal lifestyle like non-pathogenic *E. coli* [10].

To determine the genotypic characteristics of *E. coli* strains from different origins, phylogenetic analysis represents a useful and rapid method [11]. According to the combination of the three genetic markers *chuA*, *yjaA,* and DNA fragment TspE4.C2., *E. coli* strains are classified in four main phylogenetic groups, named A, B1, B2, and D [12]. Human ExPEC strains have been found to belong mainly to phylogroups B2/D, whereas commensal *E. coli* strains pertain to phylogroups A/B1 [7,13]. *E. coli* strains of phylogroup B2, genetically very similar to ExPEC, have been frequently reported in the intestine of healthy poultry and as contaminants in food of animal origin [7,8].

Commensal *E. coli* living in the gut of animals presents strains that are often extensively multidrug resistant, and therefore it has been selected as an antimicrobial resistance sentinel, as it provides valuable data and constitutes a reservoir of resistance genes [14]. To evaluate the impact on humans, other animals, and the environment, surveillance has become necessary; since 2014, the monitoring of antibiotic resistance in indicator *E. coli* from farm animals and their derived food products has been mandatory under EU legislation [15].

The physiology of *E. coli* in environmental reservoirs is poorly understood, and the knowledge of the mechanisms involved in non-host persistence is important for developing effective strategies to prevent the contamination of food products. An aspect of *E. coli* non-host persistence and survival is biofilm formation [16]. These organized structures of bacterial cells that produce a self-encasing polymer extracellular matrix provide ecologic advantages to the enclosed bacteria, including protection from environmental stresses such as temperature, pH and osmotic extremes, UV light exposure, and desiccation [17]. Furthermore, bacteria living in biofilms also exhibit enhanced resistance to cleaning and sanitation [18].

In an effort to replace current chemical disinfectants, natural substances with less environmental burden, such as essential oils (EOs), have been envisioned as an efficient and ecologically safer alternative tool to counteract microbial growth and to eradicate biofilms [19,20]. Indeed, EOs are concentrated natural plant extracts, which have proven to be good sources of bioactive compounds with antioxidant and antimicrobial properties [21]. Encouraging results for disinfection in poultry farm environments with EOs [22], in order to reduce the risk of infection for both animals and workers, have already been described.

It has been reported that EOs from different species of the genus *Satureja*, belonging to the large botanical family of *Lamiaceae,* possess remarkable antibacterial activity against several different Gram-positive and Gram-negative bacteria [21,23,24]. The volatile fraction is mainly characterized by oxygenated monoterpenes, e.g., thymol and carvacrol, whose amount can be assumed as an indicator of the antimicrobial activity [25]. SEO, as well as other essential oils and many natural active compounds, show low hydrophilicity and intrinsic dissolution rate, low absorption, poor pharmacokinetics or physical/chemical instability, and release of volatile compounds. In order to obtain better biopharmaceutical properties, and reduce doses and side effects, as well as to preserve the volatile compounds that show the main antimicrobial activity, a great effort is needed to develop suitable delivery systems. Recently, nanotechnology has had a significant impact in the field of medicine, food, cosmetics, skincare, agriculture, and broiler houses [26], and supported their improvement. Scientists have developed innovative drug delivery systems characterized by high physical–chemical stability, easy and reliable production, useful features, and low cost. Nanoparticles, liposomes, solid lipid particles, micelles, surfactant vesicles, and nanoemulsions (NEs) have been used to deliver payloads, e.g., drugs, proteins, peptides, nucleic acids, or antibiotics, with different physical–chemical properties and activities. Nanocarriers, made from organic and biocompatible materials, represent the best solution for the delivery of therapeutic agents. Nanoformulations can in fact protect the active compounds from chemical degradation and can improve their efficacy and decrease their toxicity [27,28,29]. NEs are characterized by the droplet size of the dispersed phase, ranging from 20 to 200 nm, obtained by a high energy method of preparation (e.g., sonication), and maintained in the same size range as non-diluted and diluted NEs [30]. Typically NEs contain oil, water, and an emulsifier [28], and may offer higher solubilization and improved bioavailability of poorly soluble active substances, and also a system to preserve the volatile compounds of essential oils.

Essential oil NEs have previously been described as effective antibacterial treatments [31,32,33]. The antibacterial activity of EOs against *E. coli* was considerably enhanced when they were converted into NEs, which was attributed to easier access of the essential oils into the bacterial cells [34]. Moreover, several reports have shown that the *E. coli* biofilm could be removed by EOs [35,36]. The encapsulation of essential oils in NEs improved not only the antibacterial, but also the anti-biofilm, activities of EOs [37,38].

Broiler production leads to the accumulation of various pollutants in poultry houses, including microorganisms. Besides commensal *E. coli* strains living in poultry gut, pathogenic bacteria could also represent a problem; the ability of *Salmonella* to survive after disinfection also poses a significant challenge in poultry farms. Pope and Cherry [39] observed that poultry litter treatment, composed of sodium bisulfate, reduced *E. coli* and *Salmonella* populations in broiler house litter, but it was not capable of eliminating those pathogens. Some authors have indicated that EO fogging in poultry houses improves hygiene standards, but the efficacy seems to be lower than that of conventional disinfectants. On the other hand, the use of EO vapors could improve poultry house hygiene on selected bacteria and fungi under laboratory conditions [36].

The aim of this study was to optimize and select the appropriate SEO NE formulations, and to evaluate their efficacy against *E. coli* strains from healthy chickens, grown in planktonic and sessile form, in order to develop an efficient product to be used in poultry farms to counteract microbial growth and biofilm formation. For this purpose, optimized and selected NE formulations with *Satureja* essential oil (SEO) were prepared and conveniently characterized. In order to evaluate and select the best NE formulation, in terms of composition and physical–chemical features, a pseudoternary phase diagram was developed. The selected SEO NE was shown to possess a good wettability and, due to the water content, can be easily sprayed with respect to the oil alone.

## 2. Materials and Methods

### 2.1. Plant Material, Essential Oil Extraction, and Mass Spectrometric Analysis

*Satureja montana* essential oil was obtained in a laboratory from the raw plants. As described in our previous study [33], SEO was obtained from the plants (leaves and flowers) grown at 500–600 a.s.l. in the Collepardo, Lazio region (central Italy) by the Sarandrea Marco and Co. s.r.l., Collepardo, (FR), Italy (http://www.sarandrea.it). The chemical fingerprint of the SEO was assayed by an untargeted metabolomics approach based on Fourier-transform ion cyclotron resonance (FT-ICR) mass spectrometry (MS), coupled with either electrospray ionization (ESI) or atmospheric pressure chemical ionization (APCI) ion sources, that cover (moderately) polar and less polar metabolites, respectively. A stock solution of SEO was filtered through 0.45 µm hydrophobic polypropylene Acrodisc (Sigma-Aldrich, Milan, Italy), diluted to a final concentration of 0.02 g/L in methanol (Sigma-Aldrich s.r.l., Milan, Italy), and then directly infused in an Apollo I ESI source, coupled with a Bruker BioApex 4.7 T FT-ICR mass spectrometer (Bruker Daltonics GmbH, Bremen, Germany). Methanolic 2 µM solutions of arginine and leucine-enkephalin (YGGFL, C_28_H_37_N_5_O_7_) were used as reference compounds for the assessment of mass accuracy. Tween 80 (Tw80), Hepes salt {N-(2-hydroxyethyl) piperazine-N-(2-ethanesulphonic acid)} were Sigma-Aldrich products (Sigma-Aldrich, Milan, Italy).

High-resolution ESI FT-ICR mass spectra were recorded in the *m/z* 80–1000 range in at least three replicates, with an acquisition size of 1 M. The list of *m/z* values was submitted to the free tool MassTRIX [40], considering protonated, sodiated, and potassiated ions (ESI(+)), and deprotonated and chlorinated ions (ESI(−)). The large number of generated molecular formulas were visualized by two-dimensional van Krevelen diagrams [29], and herein, based on the univocal KEGG ID obtained by the MassTrix data treatment, metabolic pathways, and the synergism among the identified metabolites, were visualized by using a donut chart and interconnection maps, respectively.

### 2.2. Pseudoternary Phase Diagram Costruction and Nanoemulsion Preparation

The pseudoternary phase diagram of SEO NEs was developed. The mixtures were prepared by combining the appropriate amounts of surfactant, oil phase, and aqueous phase (HEPES buffer, pH 7.4) in different weight ratios (Table 1), in a test tube, and were vortexed vigorously for 5 min to ensure thorough mixing. Visual inspection was made after each sample preparation. The NE formulations were prepared using Tween-80 and SEO in 5 mL of HEPES buffer (10^−2^ M, pH 7.4), in an oil/surfactant ratio of 1:1. The mixture was vortexed for about 5 min to allow the micro-emulsion formation, and then the obtained microscale droplets were sonicated for 20 min at 50 °C, using a tapered microtip operating at 20 kHz at an amplitude of 18% (Vibracell-VCX 400, Sonics, Taunton, MA, USA), to obtain the NEs. At this stage, all formulations can be sterilized by using cellulose filters (0.22 µm) in accordance with Ph. Eur.

### 2.3. NE Characterization

Droplet size distribution and the ζ-potential of the NEs were measured at the temperature of 25 °C by dynamic light scattering (DLS), using a Zetasizer Nano ZS90 (Malvern Instruments Ltd., Worcestershire, UK), equipped with a 5 mW HeNe laser (wavelength λ = 632.8 nm) and a digital logarithmic correlator.

The polydispersity index (PDI) value was also determined in order to evaluate homogeneity of the size distribution; in particular a PDI value lower than 0.3 indicates a monodisperse population.

The selected sample (number 21), included in the homogeneous phase region of pseudoternary phase diagram, was analyzed by DLS pre and post-sonication. The same sample was also observed by transmission electron microscopy after absorption onto carbon-coated copper grids. NEs were negatively stained for 10 s with 2% filtered aqueous sodium phosphotungstate adjusted to pH 7.0 and observed with a Philips 208S transmission electron microscope (FEI Company, Hillsboro, OR, USA) at 80 kV. A deep physical–chemical characterization of the selected sample was carried out in our previous work [33].

### 2.4. Stability Studies

Size measurements, by means of dynamic light scattering, were carried out before and after nebulization by a jet nebulizer (Nebula Air Liquide Medical Systems S.p.A., Bovezzo, Italy), in order to evaluate NE stability. The sample was opportunely diluted in the same buffer used for its preparation. NE size distribution was measured on a Malvern Nano ZS90 (Malvern, Worcestershire, UK) at 25 °C, with a scattering angle of 90.0°. The same apparatus was used for the evaluation of ζ-potential, using a NE preparation appropriately diluted in HEPES buffer (10^−2^ M, pH 7.4) at 25 °C. Moreover, with the same apparatus, the hydrodynamic diameter and ζ-potential of the NEs was evaluated in a temperature interval ranging from 32 °C to 20 °C, to simulate the broiler house temperature conditions.

### 2.5. Bacterial Strains

A total of thirty independent chicken *E. coli* strains, characterized for antibiotic resistance, phylogenetic group, and virulence factor presence, from a collection of characterized laboratory bacterial isolates [41], were obtained from manure samples of three representative feedlots within the Lazio region. *E. coli* ATCC 25922 was used as a control reference strain.

### 2.6. Evaluation of Microbial Biofilm Formation

Biofilm assays were conducted in 96-well polystyrene microplates: the medium used for the overnight bacterial growth was tryptic soy broth (TSB). A volume of 20 μL of diluted bacteria culture was added (OD 600 nm = 0.1) to wells filled with 180 μL of medium, and plates were incubated at 37 °C for 48 h. Subsequently, bacterial suspensions were removed by aspiration and, after washing twice with PBS, fixed by methanol (99.8% *v/v*) for 15 min. To quantify biofilm production, after the removal of methanol, wells were stained with crystal violet (2% *w/v*) for 20 min, rinsed three times with H_2_O, and eluted with 95% ethanol. Absorbance was measured at 570 nm with a microplate reader (Bio-rad Benchmark, Hercules, CA, USA). Biofilm production was classified into four groups: no biofilm, weak, moderate, and strong, according to Stepanovic et al. [42]. As a positive control for biofilm formation ability, *E. coli* LF82 strain was used [43].

The cut-off OD (ODc) was defined as three standard deviations above the mean OD of the negative control:

OD ≤ ODc = no biofilm producer
ODc < OD ≤ (2 × ODc) = weak biofilm producer
(2 × ODc) < OD ≤ (4 × ODc) = moderate biofilm producer
(4 × ODc) < OD = strong biofilm producer

### 2.7. Minimum Inhibitory Concentration (MIC) and Minimum Bactericidal Concentration (MBC)

Minimum inhibitory concentration (MIC) for the SEO was estimated by the broth micro-dilution method, consisting in serial twofold dilution, starting from 50 mg/mL to 0.05 mg/mL, using Muller Hinton Broth (MHB) (Oxoid, Basingstoke Hampshire, UK), with the addition of Tween-80 (0.02%) to enhance oil solubility. MIC–MBC was measured according to the National Committee of Clinical Laboratory Standards (CLSI, http://clsi.org/). The bacterial cultures at exponential growth were diluted to a cell density corresponding to 0.5 McFarland, and 10 μL of 10^6^ CFU/mL of each bacterial suspension was inoculated in wells. After 24 h incubation at 37 °C, the microbial growth was visually assessed. MIC value is defined as the lowest EO concentration without a visible growth. MBC is defined as the lowest concentration of EO that kills 99.9% or more of the inoculum, and is determined by sub-culturing on tryptic soy agar (TSA, Oxoid, Basingstoke Hampshire, UK) for 24 h 10 μL from each well with no visible growth.

### 2.8. Evaluation of Biofilm Inhibition

To measure the biofilm inhibition induced by the SEO and selected NEs, the growth medium was supplemented with 0.02% of Tween-80, then the SEO and NEs were twofold diluted into the medium at sub-MIC concentrations. EO and NE inhibition of cell attachment was evaluated after 48 h incubation at 37 °C. Values higher than 40% were considered significant in biofilm inhibition.

The percentage of biofilm inhibition by sub-MIC EO and NE has been calculated using the following formula [44]:

$$Biofilm\;inhibition\;\left(\%\right)=100-\frac{OD570\;sample}{OD570\;control\;}\times 100$$

### 2.9. Evaluation of Biofilm Eradication

The effect of SEO and NEs on established biofilms was evaluated after 24 h bacterial growth in polystyrene 96-well plates at 37 °C. After this incubation time, the supernatant was removed by aspiration, and replaced with medium with SEO or NEs added at sub-inhibitory concentrations and 0.02% of Tween-80. After incubation with the substances for 24 h a 37 °C, unattached bacterial cells were removed, wells were rinsed twice with PBS, and stained with crystal violet (2% *w/v*), as previously described. Absorbance was measured at 570 nm with a microplate reader (Bio-rad Benchmark, Hercules, CA, USA).

### 2.10. Scanning Electron Microscopy (SEM)

To visualize the effect of the selected SEO NEs on the morphology of bacterial strains and the inhibition of biofilm formation, SEM was performed. The NEs were added to selected different biofilm producers at 1 and ½ of relative MIC concentrations. A 1 mL sample from each tube was seeded onto glass slides in 24-wells culture plates and incubated for 48 h. Samples were then washed twice with PBS (pH 7.4) and suspended in 2.5% glutaraldehyde (*v/v*) in 0.1 M cacodylate buffer (pH 7.4). After overnight fixation at +4 °C and washing with 0.1 M cacodylate buffer, samples were post-fixed with 1% OsO_4_ in 0.1 M cacodylate buffer (pH 7.4), dehydrated in ethanol–water mixture with increasing ethanol concentrations (35%, 50%, 70%, 85%, 95%, and 100%), and dried with hexamethyldisilazane (HMDS, Sigma-Aldrich, St Louis, MO, USA) to remove fluids. Dehydrated specimens were gold-sputtered and observed by ultra-high resolution field emission gun scanning electron microscopy (FEG-SEM, FEI Company, Hillsboro, OR, USA). Secondary electron images were performed with an acceleration voltage of 20 KV. The images were processed for display using Photoshop software (Adobe Systems Inc., San Jose, CA, USA).

### 2.11. Statistical Analysis

Each experiment was performed in triplicate, and all values were reported as mean ± standard deviation (SD). The χ^2^ test with Yates’s correction for continuity was used to assess the presence of statistically significant difference between groups for discrete variables, while the Kruskall–Wallis test followed by Dunn’s post hoc pairwise test was used for continuous variables. Where necessary, the *p* values were corrected with the Benjamini–Hockberg procedure in order to account for multiple comparisons. A *p* value ≤ 0.05 was considered statistically significant.

## 3. Results

### 3.1. Oil Composition and Determination of the Role of SEO Components in Biological Pathways

**Figure 1:**
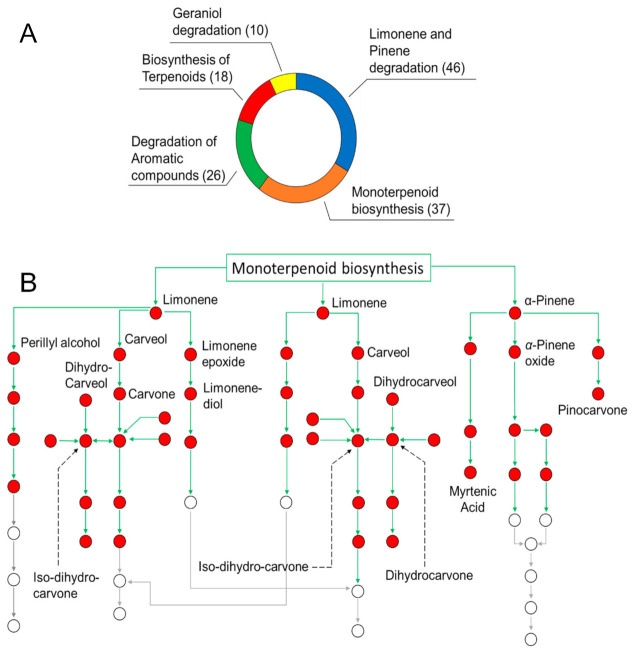
(Panel **A**): Donut chart for the most populated biological pathways in SEO. Numbers of identified hits in each path are reported in brackets. (Panel **B**): Connection network of “limonene and pinene degradation” starting from monoterpenoid biosynthesis among annotated metabolites in SEO.

As reported in our preliminary contribution [20], untargeted analyses of SEO by means of direct infusion high resolution ESI FT-ICR MS in both positive and negative ionization mode (Figure S1A,B, respectively) have allowed to identify up to 400 compounds, which belong to several phytochemical classes, such as terpenes, terpenoids, alcohols, and lipids and derivatives. Additional information on the less polar portion of SEO has been obtained by the application of APCI source (Figure S2A,B, shows the MS spectra in positive and negative mode, respectively) which highlighted the presence of less polar metabolites like borneol and camphor at *m/z* 155.0 and 153.0, respectively. Referring to the Kegg database [45], in this study a univocal ID was obtained for each compound and inserted into reference plant pathways, to evaluate the role of specific EO components. Figure 1A hows the most populated channels with the number (in brackets) of the identified compounds involved in each biochemical pathway.

As expected, the pathways engaged in the biosynthesis and degradation of monocyclic and bicyclic hydrocarbons appeared as the ones with the highest number of observed features. In particular, these reactions involve many metabolites among the most abundant components of SEO [33], including limonene, α-pinene, and carveol, in the limonene and pinene degradation pathway (map 00903, 46 hits), and linalool and geraniol, in the monoterpenoid biosynthesis path (map 00902, 37 hits). All these compounds play a key role as building blocks in plant metabolism. In general, EOs are very complex mixtures, whose components take part in interrelated routes. In particular, this is the case of p-cymene, which is able to follow two distinct routes: in fact, it can be metabolized within the degradation of aromatic compounds pathway, and/or hydroxylated to obtain thymol and carvacrol. On the other hand, some metabolites participate exclusively in a single path, as in the case of geranic acid and camphene in the geraniol degradation (map 00281), and the biosynthesis of terpenoid (map 01062), pathways, respectively. As an example, the map representing the connection network of metabolites in the limonene and pinene degradation path is shown in Figure 1B. Red dots represent the compounds annotated by FT-ICR MS in SEO [33]. The elucidation of the mutual interconnection among metabolites can be employed to better understand the mechanisms of synergism or antagonism beneath the modulation of the antibacterial activity of SEO components. Obviously, given the complexity of these phenomena, the coverage of the entire chemical composition is much more informative compared to the targeted study of SEO single components, and thus needs to be preferred. As previously described in Maccelli et al. [33], chemical analyses of both the polar and volatile constituents of SEO showed that thymol and carvacrol were the main identified oxygenated monoterpenes. Other compounds were revealed, including γ-terpinene, p-cymene, borneol, bisabolene, trans-caryophyllene, and α-pinene [33].

### 3.2. NE Design and Characterization

**Figure 2:**
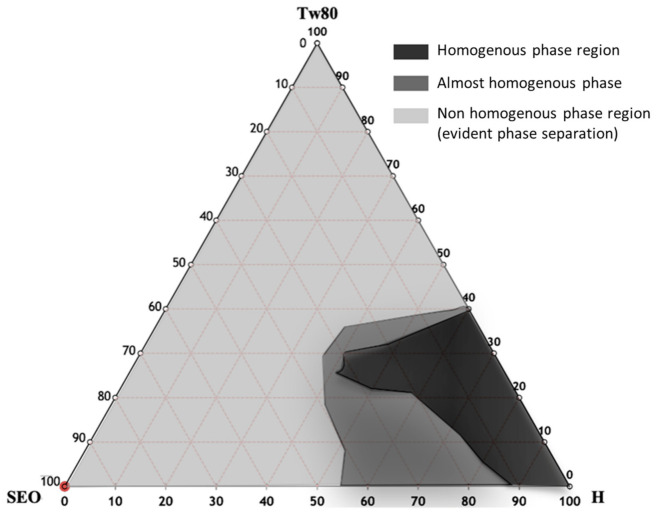
Ternary phase diagrams between SEO, Tween-80, and HEPES buffer (black colored region indicates the self-emulsification region). The resulting phases observed were the homogenous phase (black region), and two-phase region (dark and light grey).

The pseudoternary phase diagram of SEO NEs was developed, and different homogeneous phase regions were identified, in order to select the appropriate NEs in terms of hydrodynamic diameter, ζ-potential, and PDI. In Figure 2 the ternary phase diagrams of SEO with Tween-80 and HEPES buffer are shown. A homogeneous phase, according to a visual inspection, can be obtained by mixing different amounts of SEO, Tween-80, and HEPES buffer. Ternary phase diagram construction is the best way to observe the homogeneous dispersion formation by mixing these three components. This study is performed to select the optimized amounts of surfactants, oil, and HEPES buffer in the development of SEO NEs. Figure 2 highlights the presence of three different regions corresponding to homogeneous dispersions (black region) and non-homogeneous dispersions, characterized by phase separation phenomena (light grey zone). Moreover, sonication leads to the formation of monophasic dispersions for some formulations in the non-homogeneous region (dark grey zone) [26,42,46].

**Figure 3:**
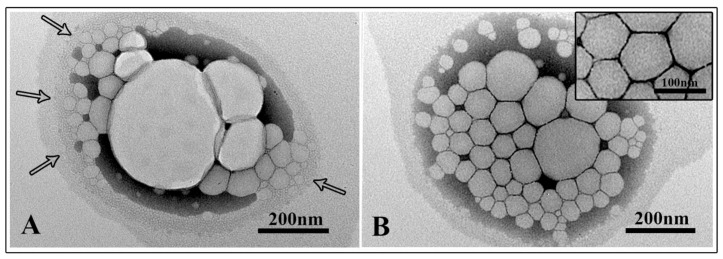
Electron microscopic observations of selected nanoemulsions (NEs) (sample 21). (Panel **A**): pre-sonication sample (arrows indicate NEs partially fused within the matrix). (Panel **B**): post-sonication sample (insert showed NEs well separated, and with a size comparable to DLS values).

To optimize the monophasic emulsions in the black region as a suitable drug delivery system (NEs), all samples were sonicated for 20 min at 50 °C. A better formulation in terms of hydrodynamic diameter, ζ-potential, and PDI was selected (Table 2). This formulation (sample 21) was also observed by TEM. Electron micrographs showed a non-homogeneous sample in the pre-sonicated NE formulation with NEs of different sizes that appeared partially fused to each other (Figure 3A, arrows) and enclosed in a matrix composed of oil and surfactant. The whole NE agglomerate, visualized in Figure 3A, showed a size comparable to the DLS value; in this case DLS was not able to discriminate among individual NEs. On the contrary, the dimensions observed for the sonicated sample confirmed the ones obtained by DLS. NEs were mainly homogeneous in size (comparable to DLS ones) and also well separated (Figure 3B and inset).

**Figure 4:**
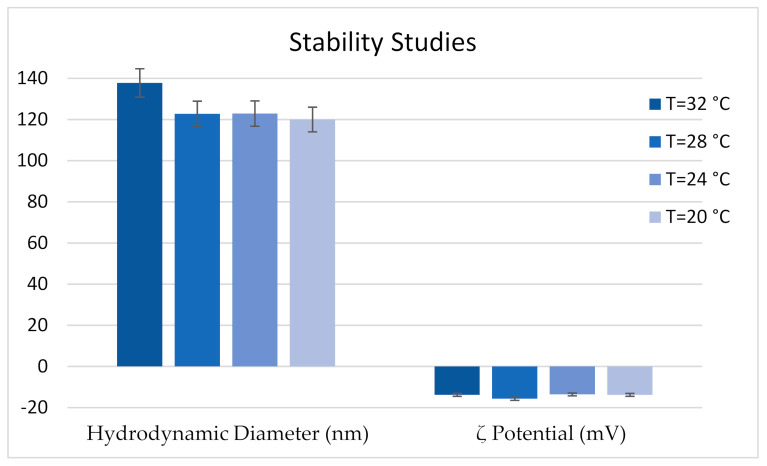
Sample 21 (post-sonication) stability in terms of hydrodynamic diameter and ζ-potential from 32 °C to 20 °C.

**Figure 5:**
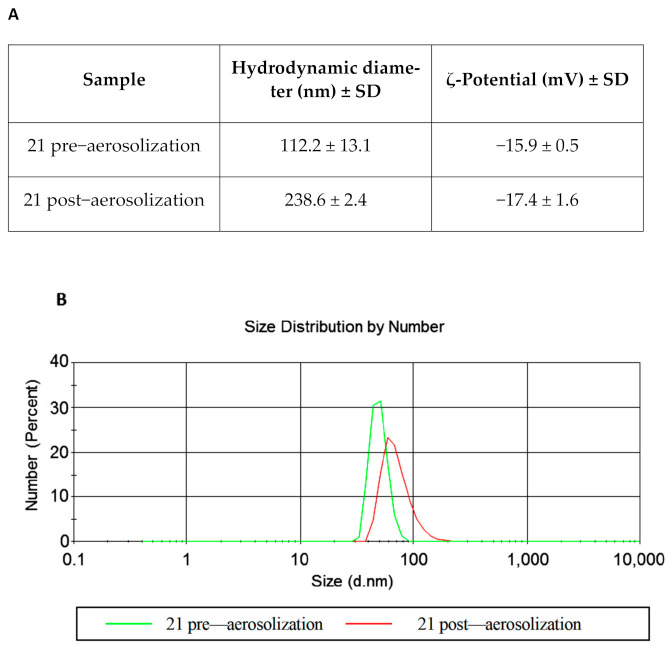
(Panel **A**): hydrodynamic diameter and ζ-potential evaluation, pre- and post-aerosolization of sample 21 (post sonication); (Panel **B**): size distribution profile pre- and post-aerosolization of sample 21 (post sonication).

Two important aspects must be taken into account to use NEs as a disinfectant to be aerosolized in a poultry farm: colloidal stability in the temperature range from 32 °C to 20 °C [36], and size and ζ-potential stability after aerosolization. Figure 4 shows that the hydrodynamic diameter and ζ-potential values of sample 21 were stable during the temperature scan experiments. Figure 5 shows that the NE integrity was preserved during the nebulization process.

### 3.3. Avian *E. coli* Strain Characterization for Biofilm Production Ability

The biofilm production ability of *E. coli* strains was classified into four groups: no biofilm, weak, moderate, and strong. As shown in Table 3, 30.0% (n = 9/30) and 27.7% (n = 8/30) of *E. coli* strains showed, respectively, a strong and moderate ability to form biofilm; 23.3% (n = 7/30) were weak biofilm producer strains, while the remaining 20.0% (n = 6/30) were totally unable to produce biofilm.

Based on data previously published [41], among strong biofilm producers some strains presented a multi-drug resistance phenotype (defined as the resistance to three or more antibiotic classes) (AV1, AV2, and AV3 strains). Interestingly, fully susceptible strains (n = 12) were also significantly associated with a strong/moderate biofilm phenotype (n = 9/12) compared to weak/no biofilm phenotype (n = 3/12). We also investigated the possible association of biofilm formation and phylotype. Interestingly, although for a low number of observations, 66.7% (n = 4/6) the *E. coli* strains belonging to the D group were strong or moderate biofilm producers [41]. *E. coli* strains belonging to the A phylogroup, the most representative phylogenetic group, were equally distributed among strong or moderate (n = 6/17 and n = 4/17, respectively), weak, or no biofilm producers (n = 2/17 and n = 5/17, respectively).

### 3.4. Antibacterial Activity of SEO and NEs against Planktonic *E. coli* Cells

As shown in Table 4, MIC values of the SEO ranged from 0.78 (n = 8/30, 26.7%) to 3.12 mg/mL (n = 13/30, 43.3%). For 66.7% (n = 20/30) of the avian *E. coli* strains the MIC and MBC values were coincident. The antibacterial efficacy of the selected optimized NE formulation was evaluated for *E. coli* strains. NE, MIC, and MBC values ranged from 0.78 to 1.56 mg/mL. Whereas 30.0% (n = 9/30) of strains exhibited a MIC value of 1.56 mg/mL, for 70.0% (n = 21/30) of strains the MIC value was 0.78 mg/mL. In nine strains the MIC and MBC were the same (0.78 mg/mL). For strong and moderate biofilm producer strains, n = 2/9 (22.2%) and n = 5/8 (62.5%), respectively, were more susceptible to NEs with respect to EO alone when grown in planktonic form. Only for the AV2 and AV8 strains was the minimal bactericidal concentration of NEs higher than the MBC of SEO. Notably, significantly higher values of MBC SEO were observed in bacterial strains unable to produce biofilm with respect to those presenting strong (*p* = 0.024) or moderate (*p* = 0.044) biofilm production. Significantly higher values of MBC SEO were observed in weak biofilm, compared to strong biofilm, producing bacterial strains (*p* = 0.042).

### 3.5. Antibiofilm Activity of SEO and NEs against Sessile *E. coli* Cells

To verify the anti-biofilm activity of sub-inhibitory concentrations of SEO and NEs, experiments of biofilm inhibition and eradication were carried out. *E. coli* avian strains (9 strong biofilm producers and 8 moderate producers) were considered.

**Figure 6:**
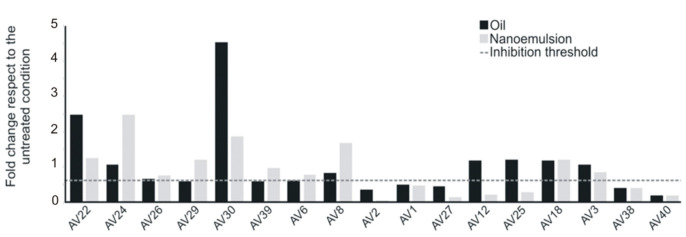
Biofilm inhibition with SEO and NEs in sub-MIC concentrations, measured through crystal violet staining.

A ≥0.4 fold decrease with respect to controls was considered to assess biofilm inhibition [44]. After 24 h incubation, sub-MIC concentrations of SEO were enough to significantly reduce biofilm production in 55.5% (n = 5/9) of the strong biofilm producers. When the SEO was in NEs this percentage rose to 77.8% (n = 7/9) (Figure 6). On the contrary, except for a few isolates, both the oil alone and in the NEs were unable to inhibit the formation of biofilm of the moderate-forming *E. coli* strains. Furthermore, for some strains, sub-MIC concentrations of EO and NEs stimulated biofilm production. Eradication of preformed biofilm was not observed in any case (data not shown).

### 3.6. Scanning Electron Microscopy Observations

**Figure 7:**
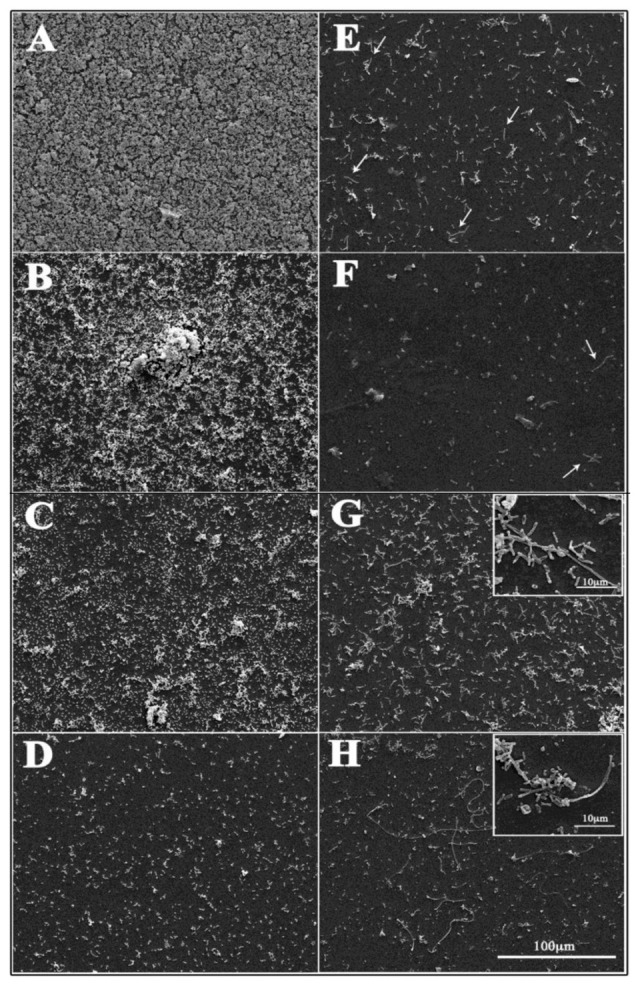
Scanning electron microscopy observations of avian *E. coli* strain biofilm formation after SEO NE treatment. Bacteria were allowed to develop biofilm on a glass slide for 48 h in the absence or presence of selected SEO NEs at sub-MIC concentrations. Micrographs show representative images of untreated (panels **A**–**D**) or SEO NEs (panels **E**–**H**) treated bacterial cells. Arrows and insets indicate elongated bacteria induced by NE treatment. Biofilm formation of LF82 (control strong biofilm producer) (**A**,**E**), AV2 (strong biofilm producer) (**B**,**F**), AV6 (moderate biofilm producer) (**C**,**G**), and AV11 (weak biofilm producer) (**D**,**H**) strains.

Bacterial biofilm production in the presence of selected NEs at sub-MIC concentrations was also evaluated by scanning electron microscopy (Figure 7). In the control groups, the untreated biofilm showed typical characteristics related to the different biofilm forming abilities of the strains. In LF82 and AV2 strains (strong biofilm producers) bacterial cells appeared tightly clustered together to form a multilayer biofilm structure (Figure 7A,B) whereas AV6 (moderate biofilm producer) and AV11 (weak biofilm producer) strains showed a smaller amount of cells attached to the substrate, and without a defined biofilm architecture (Figure 7C,D). After the addition of NEs, almost no biofilm characteristics and few bacterial cells were observed in the LF82 control strain and the strong biofilm producer AV2. Only a few elongated cells were visualized (Figure 7E,F, arrows). On the contrary, in the moderate biofilm producer, AV6, a slight decrease of attached cells was revealed (Figure 7G and inset), with much filamentous bacterial cells that were more evident in size and number in the weak biofilm producer, AV11 (Figure 7H and inset).

## 4. Discussion

The presence of *E. coli* represents an indicator of fecal and environmental contamination, and commensal animal *E. coli* strains are regarded as indicators of antimicrobial resistance [47]. Community lifestyle and standardized arrays of genetic tools have contributed to assigning to *E. coli* the role of a model organism for studying surface microbial colonization [48]. Some authors, have evidenced that avian fecal commensal *E. coli* strains are generally more able to form biofilms than avian pathogenic *E. coli* [49].

Based on our results, it would appear the 30.0% of *E. coli* strains from healthy chicken were able to produce strong biofilms on abiotic surfaces. Among the strong biofilm *E. coli* producers multi-drug resistant strains were present; furthermore, *E. coli* strains belonging to the D group, were prevalently strong or moderate biofilm producers [41].

It is believed that groups B2 and D include the majority of virulent extraintestinal *E. coli*, whilst groups A and B1 primarily represent commensal characteristics [12]. Phylogroups A and D were the most common phylogroups of *E. coli* isolated from poultry in Italy [50]. As the genetic structure of phylogroup D is more influenced by soil environments than any other phylogroup, it has been suggested that this may lead to selection for greater biofilm formers of phylogroup D *E. coli* in soil environments [51].

To reduce the use of chemical sanitizers with antimicrobial activity in the food industry, due to their negative effects, essential oils and their components have been shown to play an important role [52,53]. The antibacterial effects of EOs and their compounds have been recently assayed against bacteria, both in planktonic and sessile forms, including *E. coli* [54,55,56]. According to previous data [24,57,58] in our study, *Satureja montana* essential oil demonstrated a clear antimicrobial activity against avian *E. coli* strains, with MIC/MBC values ranging from 0.78 to 3.12 mg/mL. By scanning electron microscopy, we previously demonstrated the bactericidal action of commercial SEO on *E. coli* cell morphology, where marked damage, with irregular and collapsed cell surfaces, was observed [24]. This effect, retained in the SEO herein studied, can be possibly ascribed to the presence of oxygenated carvacrol and thymol, which are both able to disrupt the outer membrane [59]. SEO NEs exhibited higher inhibitory effects than SEO alone against planktonic *E. coli*, with MICs ranging from 0.78 to 1.56 mg/mL.

The anti-bacterial activity of our selected SEO NEs agreed with other recent studies showing that the conversion into NEs of EOs greatly improved their antimicrobial activity [60,61]. Due to the presence of lipopolysaccharide, which represents a protection system from hydrophobic compounds, it has been suggested that Gram-negative bacteria are more resistant to the essential oil treatment than Gram-positive bacteria [52]; the reduced hydrophobic property of emulsion formulations could increase the antimicrobial effect of EOs on Gram-negative bacteria.

Our results are encouraging for the application of SEO NEs, which were developed by taking into account different aspects of the optimization of the preparation. Since the surfactant and oil composition can strongly influence the NE stability and application field, to select the best surfactant/oil/water ratio a ternary phase diagram was constructed and evaluated. Due to the fundamental role of using different surfactants with different HLB (hydrophilic-lipophilic balance) values, this is the parameter that must be taken in account to prepare a stable nanoemulsion. In particular, Tween-80 (HLB = 15), with respect to Tween-65 (HLB = 10.5) (data not shown), was able to form larger monophasic emulsions areas in the ternary phase diagrams, probably due to the higher HLB value [62], which promoted the formation of o/w emulsion. The ternary phase diagram gives the relevant information on the optimum oil/surfactant/HEPES buffer ratio that has to be used for the preparation of a thermodynamically stable nanoemulsion [63]. It is well known that there are some difficulties in distinguishing nanoemulsions from microemulsions. In principle, microemulsions can be formed spontaneously by simply mixing oil, water, and surfactant together without supplying any external energy while, nanoemulsions always require the input of some external energy to convert the separate components into a colloidal dispersion. Nanoemulsion fabrication methods can be broadly categorized as either high-energy or low-energy. In this case the method employed to prepare the NEs was a high energy method (sonication).

Another parameter that could be taken in account to distinguish microemulsions from nanoemulsions is the size change after sample dilution. In particular, the NE’s size did not decrease after sample dilution. For these reasons the selected sample could be considered a nanoemulsion [64].

For anti-bacterial evaluation, the NE formulation (sample 21) was selected inside the dark zone according to the following considerations:

(i) wettability: the appropriate surfactant amount should reduce the contact angle and increase the surface wettability. Moreover, a high aqueous content could be useful to obtain a better nebulization performance;

(ii) efficacy vs toxicity evaluation [24];

(iii) physical–chemical features: the sample selected in the dark zone (homogeneous phase) showed a useful droplet size (the optimal surfactant concentration can lead to the desired particle size) and ζ-potential, as well as high stability over time at different temperature (from 32 to 20 °C).

Stability of formulations is the major problem associated with the design and development of liquid-based formulations. NEs must be evaluated in terms of physical–chemical stability. For this purpose the NE stability was evaluated at various temperatures (from 32 to 20 °C) and using various post-aerosolization processes. The results obtained by the two stability experiments, showed no significant changes in terms of hydrodynamic diameter, ζ-potential, and PDI, so it is possible to conclude that the selected NEs are thermodynamically stable between 32 and 20 °C, and after aerosolization.

The activities of the EOs reflect quite well the biological effects of the major components of the mixture [65]. However, even if present in trace amounts, several minor components of EOs enable the modulation of the biological properties [65], causing the rise of a wide range of synergism and/or antagonism effects. The antimicrobial activity of the members of the *Satureja* genus has been previously described and related to its content in secondary metabolites, including carvacrol, thymol, and terpinen-4-ol, probably through membrane damage [54]. An extensive knowledge of SEO composition and the relationship among metabolites can certainly help the comprehension of the biological activity. The same biosynthetic derivation, based on a hydroxylation processes from p-cymene [66], and therefore the structural similarity, gives to carvacrol and thymol the same mechanism of action amplified by synergism. Moreover, additional synergism was already reported between the phenolic monoterpenes and other phytochemicals identified in SEO, including several phenylpropanoids and hydrocarbons monoterpenes (α-pinene, camphene, and myrcene) [67]. Among the phenylpropanoids, the usage of FT-ICR and GC-MS revealed the presence of both eugenol and the methylated derivative (methyl-eugenol) as end-products of the phenylpropanoid biosynthesis pathway. However, the presence of the free OH seems to be exclusively involved in synergism; indeed, the greatest antimicrobial activity of eugenol (-OH) was demonstrated in relation to methyl–eugenol (-O-Me) [68]. Pei et al. [54] assumed that the synergism is due to the ability of carvacrol and thymol to disintegrate the bacterial outer membrane, thus permitting eugenol to enter the cytoplasm and react with the target protein. However, the great bioavailability of several classes of phytochemicals detected in SEO also makes possible the presence of antagonism among metabolites. Previous studies of binary mixtures of terpenes and terpenoids reported that carvone is usually antagonist towards most SEO components [69]. The connection map in Figure 1 revealed it to be a metabolic product of limonene, which conversely has never shown this type of activity. The higher content of terpenes and terpenoids vs carvone in the present SEO sample may suggest a predominant synergic effect due to limonene’s wealth in carvacrol.

D-limonene nanoemulsion inhibited *E. coli* biofilm formation through the suppression of curli and extracellular polymeric substance (EPS) production without inhibiting cell growth, and decreased swimming and swarming ability.

In our research, after 24 h of incubation, sub-MIC concentrations of SEO were enough to significantly reduce the biofilm production of 55.5% of the strong biofilm producer strains. When the SEO was in NEs this percentage rose to 70.0%. Scanning microscopic observations regarding essential oil activity on biofilm formation appeared more interesting, because they correlated to the biofilm forming ability of different strains. At sub-MIC concentrations, SEO NEs were shown to mainly inhibit the attachment to the substrate of strong biofilm producers, resulting in a poor biofilm layer; on the contrary, SEO NEs do not appear to significantly inhibit biofilm formation, but rather induce a lot of elongated bacterial cells in moderate or weak biofilm producer strains.

Biofilm formation is due to bacterium–bacterium interactions, and associations with higher organisms through intercellular communication, known as quorum sensing (QS) systems [70]. It has been suggested that D-limonene NEs inhibit QS-based virulence phenotypes in *E. coli*, including biofilm formation, curli and exopolysaccharides production, and swimming and swarming motility [70,71]. From the obtained results it can be hypothesized that SEO alone and, to a greater extent, SEO NEs, exerted an inhibitory activity on strong biofilm producers through modulation of QS molecule production or release, preventing the achievement of the threshold concentration for biofilm development. The inhibition of bacterial efflux pumps could be one of the possible mechanisms of decreased QS molecule release. It has been reported that EO from *Satureja hortensis* may act as a potential inhibitor of the *S. salivarius* and *S. acidominimas* efflux pumps [72]. Some studies have indicated that thymol and carvacrol might serve as potential sources of efflux pump inhibitor in food-borne pathogens [73]. Matsumura et al. (2011) [74] have shown that efflux pumps play important roles in biofilm formation; furthermore, employing mutant strains of *E. coli* K-12, lacking various efflux pump genes, the authors found that all the strains displayed decreased biofilm formation. As also reported by Yuan et Yuk (2019) [75], the lack of, or defects to, bacterial efflux pumps could be responsible for the different mechanism of action of SEO or SEO NEs on moderate or weak biofilm producers observed in our study. The number of bacterial cells adherent to the substrate appeared not to be significantly influenced by SEO NEs, although a different amount of elongated cells was evidenced. These morphological changes were also described by Nostro et al. (2009) [76] and Sandasi et al. (2008) [77] for staphylococcal and *Listeria* biofilms after exposure to EOs. The morphological changes of some strains after carvacrol contact were comparable to those described after treatment with other antimicrobial agents, such as antimicrobial peptides [78]. The presence of division septa in the treated cells may have been due to the effect of carvacrol on the proteins involved in cell division. Moreover, Kwon et al. (2003) [79], testing the effect of cinnamaldehyde on the morphology of *B. cereus*, found that bacterial cells appeared as elongated, filamentous structures in which the cells did not appear to be separated from one another. These modifications could also be interpreted as an adaptive response to stress.

Stress response could be also responsible for the enhancement of the biofilm formation of some moderate biofilm strains observed in our study. EOs have been observed to exert a stimulating effect on biofilm activity; an increased number of sessile cells attached to the substrate was observed in mature biofilm of *S. aureus* after exposure to low concentrations of *Origanum vulgare* EO [80]. The inductive effect of the oil occurs in the presence of sub-MICs of phenolic compounds, as a response to stressful conditions, and enhancing the biofilm formation capability of the microorganisms [38].

Unfortunately, EOs, both free and in NEs, had no activity on preformed biofilm. These results are in agreement with previous studies suggesting that EOs are able to eliminate only the cells next to the interface biofilm [81,82].

## 5. Conclusions

Non-nutritional factors such as hygiene, processing of feed ingredients, ambient temperature, animal health, and genetic makeup have an impact on animal life cycles [83]. Given the increasing restrictions imposed on poultry production in terms of food safety and the ethical aspects of husbandry, it seems appropriate to look for the use of natural substances to be applied in animal production. The main advantage of essential oils is that they do not lead to the increase of microbial resistance, and unlike disinfectants and antibiotics, oil residues are not found in the final products [84].

Improved understanding of the risk of chicken-source fecal *E. coli* need to guide the development of innovative and preventive strategies to reduce infection in poultry and subsequent food contamination. Due to the demonstrated lower cytotoxicity, with respect to essential oil alone [33], our results suggest that NEs could represent a promising strategy to counteract microbial growth and biofilm formation in poultry farming. Notably, these NEs are stable over time; after nebulization and in the time present in a poultry farm.

The anti-biofilm effect of EOs is the results of several factors, including bacterial species- and strain-dependent response, therefore combination with innovative technologies or common sanitizers can be considered a promising way to improve the effect of EOs. Natural delivery systems could represent a focal area for future research, but further studies are needed to optimize the formulations, in terms of oil content and the NE entrapment efficacy of the antimicrobial agent. Moreover, different essential oils together with different surfactants can be used and evaluated, in trying to achieve a potentiated synergic effect.

## Figures and Tables

**Table 1:** Composition of all studied formulations in the pseudoternary diagram; in bold, sample composition in the dark region.

Sample	SEO (g)	Tw80 (g)	HEPES (g)	SEO% *w/w*	Tw80% *w/w*	HEPES% *w/w*
1	0.180	0.020	0.600	22.00	3.00	75.00
2	0.180	0.020	0.800	18.00	2.00	80.00
3	0.193	0.086	0.300	33.00	16.00	51.00
4	0.193	0.086	0.450	26.00	13.00	61.00
5	0.500	0.083	0.500	38.00	6.00	56.00
6	0.180	0.020	0.400	30.00	4.00	66.00
7	0.050	0.450	0.500	5.00	45.00	50.00
8	0.193	0.086	0.150	45.00	20.00	35.00
9	0.500	0.083	0.250	60.00	10.00	30.00
10	0,500	0.083	0.250	46.00	8.00	46.00
11	0.200	0.266	0.200	30.00	40.00	30.00
12	0.180	0.020	0.200	45.00	5.00	50.00
**13**	**0.072**	**0.142**	**0.500**	**10.00**	**20.00**	**70.00**
**14**	**0.083**	**0.250**	**0.500**	**10.00**	**30.00**	**60.00**
**15**	**0.050**	**0.450**	**1.000**	**2.50**	**22.50**	**75.00**
**16**	**0.225**	**0.225**	**0.300**	**30.00**	**30.00**	**40.00**
**17**	**0.225**	**0.225**	**0.600**	**21.50**	**21.50**	**57.00**
**18**	**0.200**	**0.266**	**0.400**	**23.00**	**31.00**	**46.00**
**19**	**0.050**	**0.450**	**0.500**	**3.00**	**30.00**	**67.00**
**20**	**0.200**	**0.266**	**0.200**	**19.00**	**25.00**	**56.00**
**21**	**0.098**	**0.098**	**5.000**	**2.00**	**2.00**	**96.00**

**Table 2:** Hydrodynamic diameter, ζ-potential, and polydispersity index (PDI) values of sample 21 pre- and post-sonication.

Sample 21	Hydrodynamic Diameter (nm) ± SD	ζ-Potential (mV) ± SD	PDI ± SD
Pre–sonication	816.3 ± 90.0	−15.9 ± 0.61	0.56 ± 0.38
Post–sonication	112.2 ± 13.1	−15.9 ± 0.55	0.22 ± 0.12

**Table 3:** *E. coli* strains: biofilm formation ability. See ref. [41]. Multi drug resistance (MDR) ≥ 3 different antibiotic classes.

	Number (%)	Strains	Antibiotic Resistance	Phylogenetic Group
STRONG BIOFILM PRODUCERS	N = 9/30 (30)	AV1	MDR	A
		AV2		D
		AV3		D
		AV12	SU,TET	B1
		AV18	SU	A
		AV25	FULL SENSITIVE	A
		AV27		B1
		AV38		A
		AV40		A
MODERATE BIOFILM PRODUCERS	N = 8/30 (27)	AV6	GM,KM,SM,TB	A
		AV8	GM,SM,SU	A
		AV22	GM	D
		AV24	FULL SENSITIVE	A
		AV26		D
		AV29		A
		AV30		A
		AV39		B1
WEAK BIOFILM PRODUCERS	N = 7/30 (23)	AV5	MDR	A
		AV7		A
		AV11	GM,SU	B1
		AV23	GM	D
		AV17	KM	B1
		AV21	SM	B1
		AV34	FULLSENSITIVE	B1
NO BIOFILM PRODUCERS	N = 6/30 (20)	AV4	MDR	A
		AV14	TET	A
		AV15	TET	A
		AV33	FULL SENSITIVE	D
		AV35		A
		AV37		A

**Table 4:** Minimum inhibitory concentrations (MICs) and minimum bactericidal concentrations (MBCs) comparison between SEO and NEs.

	STRAINS	MIC SEO ^1^	MBC SEO ^1^	MIC Nes ^1^	MBC Nes ^1^
STRONG BIOFILM PRODUCERS	AV1	3.12	3.12	0.78	1.56
	AV2	0.78	0.78	0.78	1.56
	AV3	1.56	1.56	1.56	1.56
	AV12	1.56	3.12	1.56	1.56
	AV18	1.56	1.56	1.56	1.56
	AV25	0.78	1.56	0.78	1.56
	AV27	1.56	1.56	0.78	0.78
	AV38	0.78	1.56	0.78	0.78
	AV40	0.78	1.56	0.78	0.78
MODERATE BIOFILM PRODUCERS	AV6	3.12	3.12	0.78	0.78
	AV8	1.56	0.78	0.78	1.56
	AV22	3.12	3.12	0.78	1.56
	AV24	1.56	1.56	1.56	1.56
	AV26	0.78	1.56	0.78	0.78
	AV29	1.56	1.56	0.78	0.78
	AV30	0.78	1.56	1.56	1.56
	AV39	3.12	3.12	0.78	1.56
WEAK BIOFILM PRODUCERS	AV5	3.12	3.12	1.56	1.56
	AV7	1.56	1.56	0.78	1.56
	AV11	3.12	3.12	0.78	1.56
	AV23	0.78	3.12	0.78	0.78
	AV17	3.12	3.12	1.56	1.56
	AV21	0.78	3.12	1.56	0.78
	AV34	3.12	3.12	0.78	1.56
NO BIOFILM PRODUCERS	AV4	3.12	3.12	0.78	1.56
	AV14	1.56	3.12	0.78	0.78
	AV15	3.12	3.12	1.56	1.56
	AV33	3.12	3.12	0.78	1.56
	AV35	3.12	3.12	0.78	1.56
	AV37	3.12	3.12	0.78	0.78

^1^ expressed in mg/mL.

## Data Availability

The data presented in this study are available upon request.

## Supplementary Materials

The following are available online at https://www.mdpi.com/1999-4923/13/2/134/s1, Figure S1: ESI FT-ICR full scan mass spectra for SEO in positive (panel A) and negative (panel B) polarity mode. The inserts show the presence of several SEO components, belonging to lipids, fatty acids and terpenoids (see ref. [20] for metabolites annotations). Figure S2: APCI-MS spectra for SEO in positive (panel A) and negative (panel B) polarity mode in a range between 80 and 400 Da (see ref. [20] for metabolites annotations).
