# Supplementary material for: Nanoemulsions of Satureja montana Essential Oil: Antimicrobial and Antibiofilm Activity against Avian Escherichia coli Strains
Source: Pharmaceutics. 2021 Jan 21;13(2):134. doi: 10.3390/pharmaceutics13020134 (PMC7909762; doi:10.3390/pharmaceutics13020134)
Supplement: Supplementary file 1 [file pharmaceutics-13-00134-s001.pdf]

pharmaceutics MDPI

Supplementary Materials: Nanoemulsions of Satureja montana Essential Oil: Antimicrobial and Antibiofilm Activity against avian Escherichia coli Strains

Federica Rinaldi, Linda Maurizi, Antonietta Lucia Conte, Massimiliano Marazzato, Alessandro Maccelli, Maria Elisa Crestoni, Patrizia Nadia Hanieh, Jacopo Forte, Maria Pia Conte, Carlo Zagaglia, Catia Longhi, Carlotta Marianecci, Maria Grazia Ammendolia and Maria Carafa

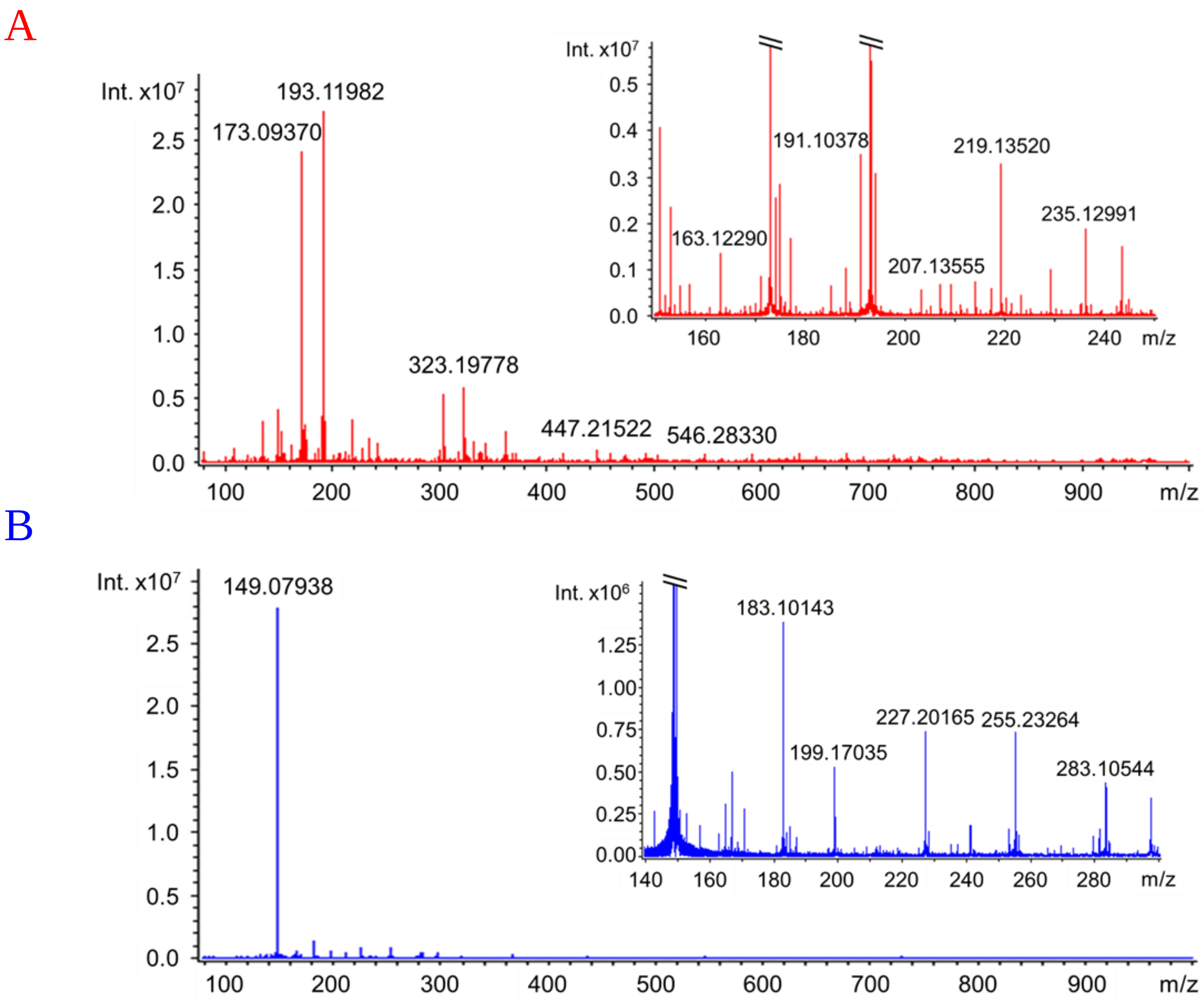

Figure S1. ESI FT-ICR full scan mass spectra for SEO in positive (panel **A**) and negative (panel **B**) polarity mode. The inserts show the presence of several SEO components, belonging to lipids, fatty acids and terpenoids [see ref. 20 for metabolites annotations].

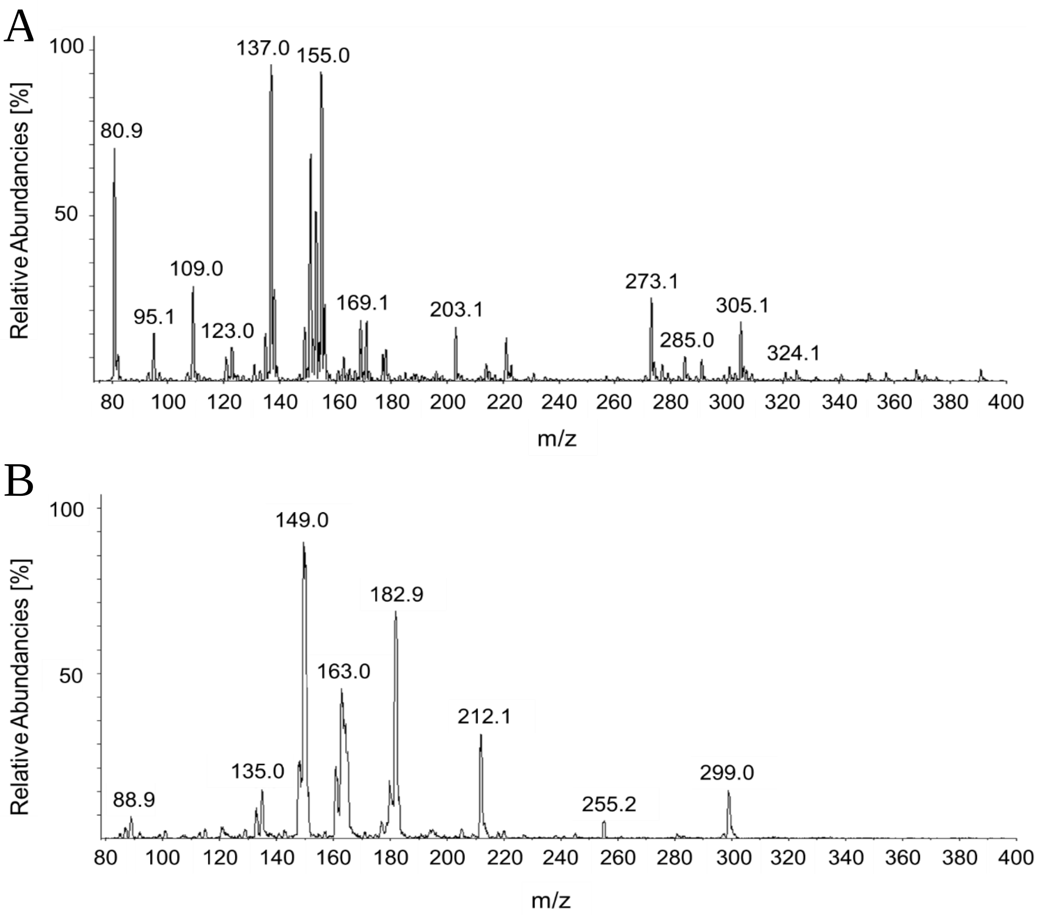

Figure S2. APCI-MS spectra for SEO in positive (panel **A**) and negative (panel **B**) polarity mode in a range between 80 and 400 Da [see ref. 20 for metabolites annotations].
